# An Index of Non-Linear HRV as a Proxy of the Aerobic Threshold Based on Blood Lactate Concentration in Elite Triathletes

**DOI:** 10.3390/sports10020025

**Published:** 2022-02-18

**Authors:** Bruce Rogers, Sander Berk, Thomas Gronwald

**Affiliations:** 1 College of Medicine, University of Central Florida, 6850 Lake Nona Boulevard, Orlando, FL 32827-7408, USA; 2 Dutch Triathlon Federation, Papendallaan 49, 6816 VD Arnhem, The Netherlands; sander.berk@triathlonbond.nl; 3 Institute of Interdisciplinary Exercise Science and Sports Medicine, MSH Medical School Hamburg, University of Applied Sciences and Medical University, Am Kaiserkai 1, 20457 Hamburg, Germany; thomas.gronwald@medicalschool-hamburg.de

**Keywords:** heart rate variability, lactate threshold, DFA a1, training intensity distribution, polarized training

## Abstract

A non-linear index of heart rate (HR) variability (HRV) known as alpha1 of Detrended Fluctuation Analysis (DFA a1) has been shown to change with increasing exercise intensity, crossing a value of 0.75 at the aerobic threshold (AT) in recreational runners defining a HRV threshold (HRVT). Since large volumes of low-intensity training below the AT is recommended for many elite endurance athletes, confirmation of this relationship in this specific group would be advantageous for the purposes of training intensity distribution monitoring. Nine elite triathletes (7 male, 2 female) attended a training camp for diagnostic purposes. Lactate testing was performed with an incremental cycling ramp test to exhaustion for the determination of the first lactate threshold based on the log–log calculation method (LT1). Concurrent measurements of cardiac beta-to-beat intervals were performed to determine the HRVT. Mean LT1 HR of all 9 participants was 155.8 bpm (±7.0) vs. HRVT HR of 153.7 bpm (±10.1) (*p* = 0.52). Mean LT1 cycling power was 252.3 W (±48.1) vs. HRVT power of 247.0 W (±53.6) (*p* = 0.17). Bland–Altman analysis showed mean differences of −1.7 bpm and −5.3 W with limits of agreement (LOA) 13.3 to −16.7 bpm and 15.1 to −25.6 W for HR and cycling power, respectively. The DFA a1-based HRVT closely agreed with the LT1 in a group of elite triathletes. Since large volumes of low-intensity exercise are recommended for successful endurance performance, the fractal correlation properties of HRV show promise as a low-cost, non-invasive option to that of lactate testing for identification of AT-related training boundaries.

## 1. Introduction

Knowledge of exercise intensity boundaries is important for guidance in athletic training and diagnostics. Various models of training intensity distribution have been proposed (polarized, threshold and pyramidal) but all share one aspect in common, that the majority of training is to be performed in a low-intensity zone range [1]. In the typical three-zone model, the upper low-intensity limit is felt to be regarded as the aerobic threshold (AT), represented by either the first lactate (LT1) or ventilatory threshold (VT1) [2]. Although an incremental ramp test with gas exchange monitoring can be performed to determine the VT1, many athletic training centers utilize progressive constant cycling power intervals with lactate testing to determine the AT using the LT1. Although, on first glance, this would seem a simple endeavor, several issues make the concept more complex. Importantly, there does not appear to be universal agreement on what concept defines the LT1 [3]. Some investigators have used a fixed value of 2 mmol/L, while others use a fixed value above baseline of 0.5 or 1 mmol/L. Other options include logarithmic plotting of lactate against cycling power or heart rate (HR) [4]. This could lead to variable results of a training boundary obtained by one measure or another.

Besides the confusion surrounding test definition, lactate testing is invasive, relatively costly and generally requires additional personnel to perform the test while the athlete is exercising. Therefore, alternate means of determining the AT have been evaluated over the years including modalities related to various heart rate variability (HRV) indexes [5,6,7]. However, despite initial appeal, general use for the purpose of low-intensity training guidance has not occurred for various reasons [8,9]. Recently, HRV monitoring during the exercise session has received a resurgence in attention as a method of measuring the AT [8]. In a group of recreational runners, the AT was found to closely match that of a HRV threshold (HRVT) derived from a non-linear HRV index of fractal correlation properties determined by alpha1 of Detrended Fluctuation Analysis (DFA a1) [9]. As exercise intensity rises, the DFA a1 declines from values near 1, which represent the well-correlated fractal behavior of the cardiac beat-to-beat pattern, passing a value of 0.75 at the AT, then reaching uncorrelated, random behavior at intensities past the AT. Potential advantages of this approach to estimating the AT include cost, easy availability of HR monitoring devices and its non-invasive nature. However, since initial validation of this approach was performed by comparing the HRVT to the AT as represented by the VT1 derived from gas exchange during treadmill running, potential agreement with an alternative method such as the LT1 evaluated during an incremental cycling stage ramp is unknown and should be addressed.

In order to show that the HRVT is a robust, reliable surrogate for the AT, evaluation in certain population subsets would also be helpful. For instance, this marker may be applicable in a group possessing average fitness abilities but loses validity with elite endurance athletes. Since the elite endurance athlete typically performs large amounts of training in the low-intensity zone, validation of the HRVT principle in these individuals would be especially beneficial. Recent observational analysis has shown that the volume of low-intensity training performed by competitive long-distance runners [10] as well as the high training volume performed by recreational half marathon runners [11] is related to their future performance. Therefore, given the need for accurate delineation of the low-intensity boundary in elite athletes for training and testing purposes, the intent of this report is to explore the relationship of the DFA a1-based HRVT with that of the LT1, obtained at an athletic team assessment camp.

## 2. Methods

### 2.1. Participants

Nine elite triathletes (7 male, 2 female) of various countries of origin were recruited from a triathlon assessment training camp. Current competition category ranged between national and international levels. Average age was 24 years (±4), body weight 69 kg (±9), height 175 cm (±10), weekly training volume 20 h (±3) and $\dot{\text{V}}$O_2MAX_ 67 mL/kg/min (±7). This study was conducted during routine diagnostic procedures in a training camp and no additional equipment was used for data collection and no further procedures were performed. Physiologic testing was performed at the beginning of the camp. Athletes had not performed any recent high-intensity exercise and were deemed well rested by the coaching staff. All participants were informed about the study procedures and objectives. They provided written informed consent according to the ethical guidelines in accordance with the institutional review board and the guidelines of the Helsinki World Medical Association Declaration.

### 2.2. Exercise Testing Protocol

An incremental cycling stage test until voluntary exhaustion was performed with a Cyclus2 ergometer (RBM elektronik-automation GmbH, Leipzig, Germany) with a freely chosen cadence between 80 and 90 rpm. The test protocol for men consisted of a starting cycling power of 90 W (watts), with an incremental rise of 30 W every 3 min. For women, the starting cycling power was 75 W, with an incremental rise every 3 min of 25 W. Machine calibration was performed in accordance with manufacturer recommendations. Ambient temperature (14 to 17 °C), altitude of 45 m and meal timing were similar for all participants. Both caffeine and alcohol consumption were avoided for 24 h pretesting. There was no tobacco usage in any participant.

### 2.3. Lactate Testing

All lactate samples were measured with the Lactate Pro2 (Arkray KDK, Kyoto, Japan) between 30 and 40 s before the end of each ramp stage, except the last sample, which was taken directly after completion of the test (10–15 s after cessation). Determination of the first lactate threshold was performed using automated testing software [4], using logarithmic plotting of lactate vs. either cycling power or HR.

### 2.4. RR Measurements and Calculation of DFA a1-Derived HRVT

A Polar H10 (Polar Electro Oy, Kempele, Finland) HR monitoring device (HRM) was used to detect RR intervals in 7 individuals, a Pioneer HRM (Pioneer Electronics, Torrance, CA, USA) Inc was used in one individual and a Garmin HRM (Garmin Inc, Olathe, KS, USA) in another due to individual preferences. All RR data were recorded with a Garmin 530 cycling computer (Garmin Inc., Olathe, KS, USA) and then imported into Kubios HRV Software Version 3.4.3 (Biosignal Analysis and Medical Imaging Group, Department of Physics, University of Kuopio, Kuopio, Finland). Kubios preprocessing settings were set to the default values including the RR detrending method which was kept at “Smoothness priors” (Lambda = 500) [12]. DFA a1 window width was set to 4 ≤ *n* ≤ 16 beats. The RR series was then corrected by the Kubios “automatic method” and relevant HRV parameters exported as text files for further analysis. Artifact levels measured by Kubios HRV were below 5%. This limit was previously shown to have minimal effect on the HRVT [13]. DFA a1 was calculated from the RR data series using 2 min time windows with repeat computation every 5 s throughout the test (time-varying method—window width = 2 min, grid interval = 5 s). Two-minute time windowing was chosen to achieve a sufficient minimal beat count [14]. For the detection of HRVT, a DFA a1 value of 0.75 was selected based on previous study in recreational athletes [9]. This value is also the midpoint between a fractal, well-correlated behavior of the HR time series of 1.0 (seen with very light exercise) and an uncorrelated value of 0.5 which represents random behavior (seen with high-intensity exercise) [8]. Plotting of DFA a1 vs. HR was then performed, generally showing a stable area above 1.0 at low work rates, a rapid, near linear drop reaching below 0.5 at higher intensity, then plateauing without major change. The procedure used to indicate at what level of cycling intensity as HR the DFA a1 would cross a value of 0.75 has been detailed previously [9]. Cycling power at DFA a1 = 0.75 (cycling power at HRVT) was calculated from the 180 s average cycling power preceding the time DFA a1 reached 0.75.

### 2.5. Statistics

Statistical analysis was performed for: HR and cycling power at LT1 derived from lactate testing; HR and cycling power at HRVT derived from DFA a1. Standard statistical methods were used for the calculation of means and standard deviations (SD). Normal distribution of data was checked by Shapiro–Wilk’s test. The agreement with LT1 parameters was assessed using linear regression, Pearson’s r correlation coefficient, intraclass correlation coefficient (ICC), Lin’s concordance correlation coefficient (CCC), standard error of estimate (SEE), and Bland–Altman plots with limits of agreement [15]. All Bland–Altman plots were assessed for proportional bias. The size of Pearson’s r correlations was evaluated as follows: 0.3 ≤ r < 0.5 low; 0.6 ≤ r < 0.8 moderate; and r ≥ 0.8 high, [16] The paired *t*-test was used for comparison of LT1 vs. HRVT for both cycling power and HR. For all tests, the statistical significance was accepted as *p* ≤ 0.05. Analysis was performed using Microsoft Excel 365 with Real Statistics Resource Pack (Release 6.8) and Analyse-it software (Version 5.66).

## 3. Results

### Lactate Threshold and HRVT Comparison

**Figure 1:**
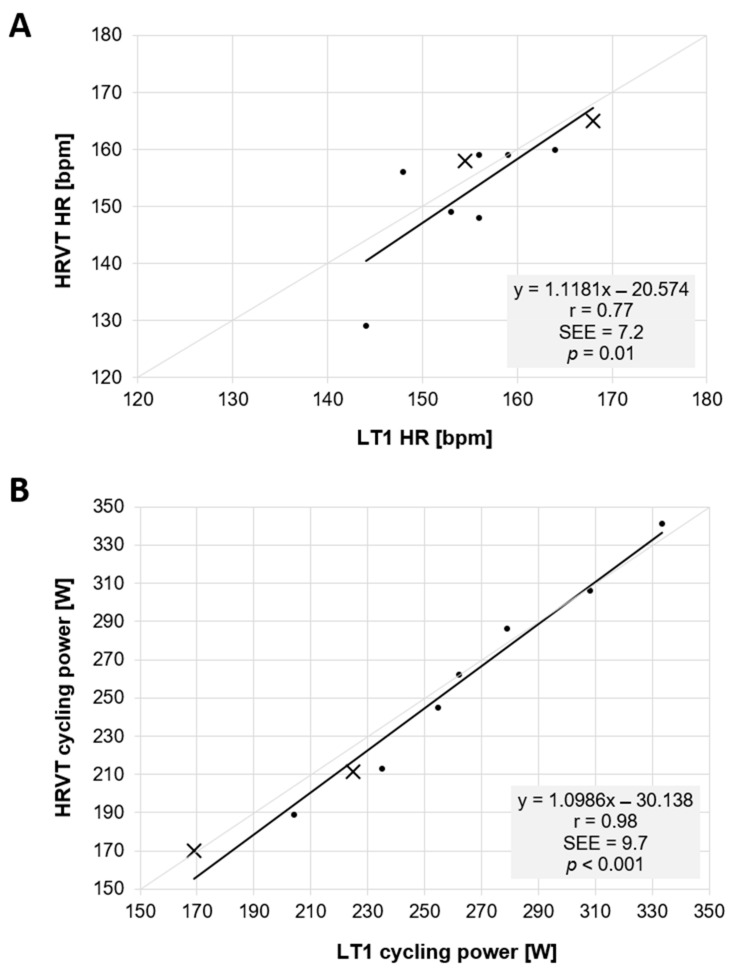
Regression plots for all participant data. (**A**): Values of LT1 vs. HRVT for HR in bpm. (**B**): Values of LT1 vs. HRVT for cycling power in W. Bisection lines in light gray. SEE: standard error of estimate; r: Pearson’s r. Points symbolized by X represent female participants.

**Figure 2:**
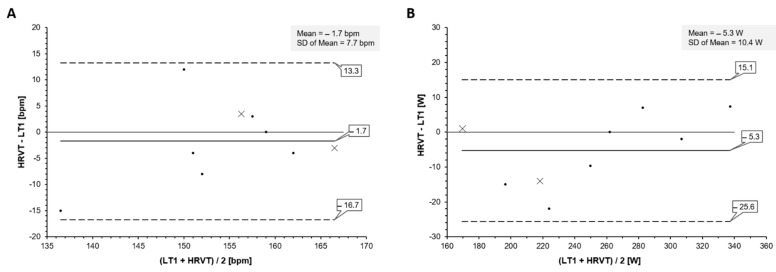
Bland-Altman Plot of LT1 vs. HRVT for all participants. (**A**): Values of LT1 vs. HRVT for HR in bpm. (**B**): Values of LT1 vs. HRVT for cycling power in W. Center line in each plot represents the mean difference between each paired value, the top and bottom lines are 1.96 standard deviations from the mean difference. Points symbolized by X represent female participants.

Mean LT1 HR for all 9 participants was 155.8 bpm (±7.0) vs. HRVT HR of 153.7 bpm (±10.1) (*p* = 0.52). Mean LT1 cycling power was 252.3 W (±48.1) vs. HRVT cycling power of 247.0 W (±53.6) (*p* = 0.17). Regression plots are shown in Figure 1 for HR and cycling power comparisons. Bland–Altman analyses for comparisons of HR and cycling power are shown in Figure 2. Mean differences were −1.7 bpm and −5.3 W with LOA of 13.3 and −16.7 bpm for HR and of 15.1 and −25.6 W for cycling power; no proportional bias was found (HR: r = 0.38 *p* = 0.31; cycling power: r = 0.55 *p* = 0.11). All data were normally distributed (All W > 0.90, *p* > 0.31). ICC (1,1) between LT1 HR and HRVT HR was 0.69 (95% confidence limits 0.14 to 0.92) and was 0.98 (95% confidence limits 0.91 to 0.99) for cycling power. CCC between LT1 HR and HRVT HR was 0.66 (95% confidence limits 0.11 to 0.90) and was 0.98 (95% confidence limits 0.92 to 0.99) for cycling power.

## 4. Discussion

This study aimed to confirm the association of the DFA a1-based HRVT with that of the LT1, an established marker of the AT [3] in a demographic consisting of elite male and female triathletes using a cycling stage ramp test. The results show clear relationships between the LT1 calculated by the log–log method and HRVT for both HR and cycling power. This was supported by linear regression, with Pearson’s r of 0.77 and 0.98 for HR and cycling power, respectively. There was no difference between mean values of LT1 and HRVT by paired t testing. Bland–Altman analysis showed minimal mean differences that were felt to be acceptable for the purpose of exercise and training prescription. Although a previous report found a good concordance between the HRVT and VT1 assessed by gas exchange, that study was performed in recreational runners using a treadmill test with research grade ECG monitoring [9]. Given the potential value of an alternate non-invasive marker for the AT, further investigation into varied user populations, alternate recording devices and exercise modalities are warranted before widespread usage. The participants evaluated here did represent an elite class of triathlete including several national team members. Weekly training volumes and $\dot{\text{V}}$O_2MAX_ measurements were in agreement with this and well above typical recreational levels [17]. Since elite endurance sport participants are frequently the subjects of training intensity distribution research, positive confirmation of the HRVT equivalence to the LT1 could be helpful with non-invasive zone boundary identification and enforcement.

The underlying mechanism for DFA a1 behavior during exercise appears to be related to the antagonistic behavior of the sympathetic and parasympathetic branches of the autonomic nervous system on the sinoatrial node [18] as well as other potential factors [19]. As work intensity rises there is a withdrawal of the parasympathetic component and enhancement of the sympathetic component with the net result being that of a decline of DFA a1 as well as other HRV parameters. However, as opposed to conventional HRV indexes that rely on a nadir to determine the AT, DFA a1 appears to pass a specific dimensionless value of 0.75. Therefore, asymptotic curve interpretation and calibration to metabolic parameters such as a lactate or ventilatory threshold is unnecessary. This has obvious advantages in both retrospective HRV analysis and real-time monitoring of exercise demands. The reasoning behind choosing a DFA a1 value of 0.75 as a focus for VT1 transition was based on several factors [8,9]. Inspection of prior DFA a1 incremental ramp studies appeared to indicate that the aerobic threshold was situated near this area [8]. In addition, this value represents the midpoint between correlated fractal patterns seen with low intensity loads (1.0) and uncorrelated random patterns seen at intensity domains past the aerobic threshold (0.5) [8]. Additional studies in other demographic groups also appear to support the 0.75 HRVT association with the gas exchange-derived VT1 [20,21]. With respect to DFA a1 activity in elite athletes, it is certainly plausible that this group could exhibit distinct differences from inactive or recreational cohorts based on factors such as cardiac remodeling [22,23] and/or altered vagal tone [24]. The concordance between the HRVT to that of the AT in both elite and non-elite populations is reassuring to the hypothesis that the DFA a1 threshold concept is widely applicable. The HRVT can also be considered as a separate concept within the theoretical framework [8] of different organismic regulation patterns.

## 5. Limitations and Future Directions

Although the derivation of the HRVT is relatively straightforward, both the definition and calculation of the LT1 are subject to numerous views (i.e., rise of either 0.5 or 1.0 mmol/L, a fixed 2.0 mmol/L threshold, etc.) [3,25,26,27]. As one of several established methods, even logarithmic transformation has interpretive options that could lead to slightly different results for cycling power or HR [25]. Therefore, comparison of the HRVT to the various LT1 concepts could be performed in future investigations of larger sample size. Scant data on DFA a1 behavior are available in female athletes and further study is needed in that population. Fortunately, in this study, two participants were female and had results similar to the entire group. A possible limitation of this study is the heterogeneity of HRM devices. Although they are all share similar form factors, it is certainly possible that unique device bias could be present. A strength of this study is that artefact presence in the included data was below 5%, which has been shown to have minimal effect on the HRVT [13]. In the future, devices with a chest belt form factor with single-lead ECG functionality would be advantageous for both evaluation of artefact type, manual correction of artefact as well as arrythmia identification [28]. It is also feasible that the DFA a1 measurement be integrated into smartwatch or smartphone monitoring devices for real-time intensity and zone 1 enforcement. Several new implementations of DFA a1 monitoring via adapted open-source python packages have recently emerged, making this idea closer to attainment [29]. Although group sample size was somewhat less than optimal at nine individuals, this is not unexpected given the exclusive nature of high-level athletic training populations. Finally, while participant training status was felt not to be overreached, further investigations into using changes in DFA a1 behavior as a measure of endurance exercise fatigue appear promising based on recent data [30].

## 6. Practical Applications

The intensity region where DFA a1 declines through a value of 0.75 (HRVT) can be used to delineate the AT in terms of HR and cycling power during an incremental cycling ramp. The agreement between the HRVT and LT1 was similar to previous VT1 and LT1 comparisons [24]. This information can be used for both measurement of current fitness status as well as for regulation and monitoring of low-intensity training distribution. Further extension of the HRVT hypothesis was confirmed in an elite group of endurance athletes.

## 7. Conclusions

A heart rate variability threshold based on DFA a1, a non-linear measure of fractal correlation properties, was closely associated with the first lactate threshold in a population of elite male and female triathletes during a cycling ramp stage test. Given the importance of identifying the upper boundary for low-intensity zone training for endurance-type sports, the DFA a1-related threshold demonstrates an exciting low-cost, non-invasive option to that of lactate testing. In addition, due to its dimensionless nature and agreement with the aerobic threshold, future use on a retrospective or real-time basis to enforce training intensity distribution guidelines is possible.

## Data Availability

The raw data supporting the conclusions of this article will be made available by the authors, without undue reservation.
